# Supplementary figures and images for: Comparative Transcriptomics Identify Key Pituitary Circular RNAs That Participate in Sheep (Ovis aries) Reproduction
Source: Animals (Basel). 2023 Aug 25;13(17):2711. doi: 10.3390/ani13172711 (PMC10486758; doi:10.3390/ani13172711)

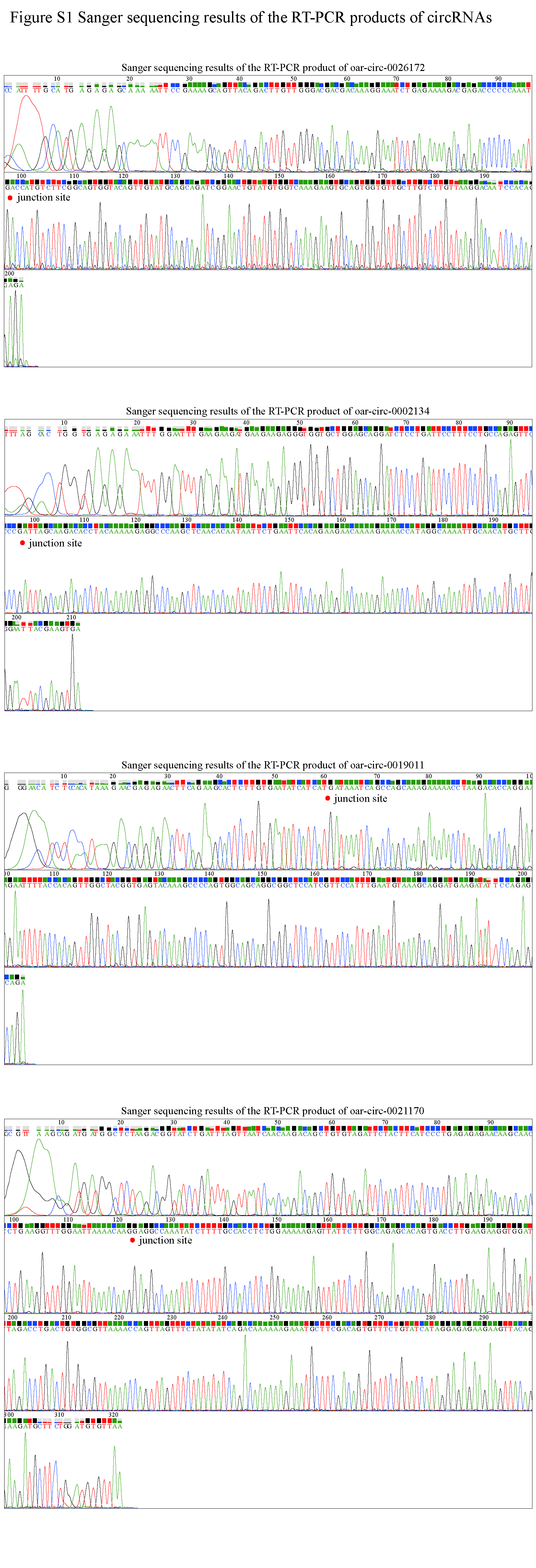

Supplement: Supplementary file 1 [file animals-13-02711-s001.zip › animals-2528678-supplementary/Figure S1 Sanger sequencing results of the RT-PCR products of circRNAs.tiff]
